# Editorial

**Published:** 1863-09

**Authors:** 


### Editorial.

ALCOHOLIC STIMULANTS AS PROPHYLACTICS.—In the department of "Selections," for the present number, will be found a letter from JAMES BRYAN, M.D., Surgeon of U.S.V. We republish it principally for the purpose of calling attention to his remarks in relation to the efficacy of vegetable acids as preventives of diarrhœa and bowel-affections during the warm season of the year. We have uniformly used these acids, more especially the acetic, freely during the summer months, for the past fifteen years. We take them with food at meal time, very rarely drinking anything whatever between meals. During all that time, though daily undergoing fatigue, and often without rest at night, we have suffered no attack of diarrhœa that lasted over twelve hours. A teaspoonful of good acetic acid vinegar with a little sugar, in a tumblerful of cold water, makes a very palatable drink; giving a pleasant and healthy tone to both the gastric and cutaneous surfaces, and, if taken at meals, promotes digestion. During the heat of summer—when the skin is eliminating an excess of perspiration, holding in solution the acid salts of the blood, a plentiful supply of vegetable acids is necessary to preserve a healthy condition of that fluid. I regret that Dr. BRYAN'S letter confirms, what indeed we had abundant evidence of before, namely, that large numbers of the officers of the army, and even of the Medical officers, habitually carry with them and use, alcoholic drinks, especially what they call "good old Bourbon whiskey," as a supposed prophylactic against bowel-affections and malarial diseases. How so many men of science, and of high professional attainments, can still cling to the use of a class of articles, whose effects in depressing the vital properties of the tissues, and retarding a healthy metamorphosis or change of structure in the natural nutrition and disintegration taking place throughout the living system; as demonstrated by every well devised physiological experiment on the subject; by investigations in morbid anatomy; and by abundant observation in social and professional life; is a mystery to all who have not studied closely the force of popular maxims and habits when aided by the fascination of a moderate cerebral exhileration. That the presence of whiskey, or any other alcoholic drink, in the blood will retard the absorption of malaria, or any other poison, by retarding organic or atomic changes, may be true. But the simple postponement in the action of noxious agents thus affected, is very much more than counterbalanced by the general depression of vital power by such interference with the atomic changes, and consequent healthy eliminations. Hence, as Dr. BRYAN very truly remarks, when persons using alcoholic drinks are attacked with disease, they are much less easily managed, and their disease much more persistent and dangerous than those affecting persons who entirely discard the use of such drinks. How much longer will the members of the Medical Profession entertain the absurd idea that the *retardation* of *metamorphosis* produced by alcoholic beverages, is equivalent to an increase of healthy nutrition?

---

SURGEON-GENERAL HAMMOND.—This officer, rendered notorious, if not famous, for his *raid* against calomel and tartar emetic, has been removed from his high position, and ordered to report for duty in the Department of the Gulf, under Gen. Banks. We hope his successor will have common sense, if no other qualification.

RESIGNATION.—Prof. H. H. Childs, of Pittsfield, Mass., one of the principal founders of the Berkshire Medical College, has recently resigned the chair of "Obstetrics and Diseases of Women and Children," in that Institution. Dr. Childs has attained an advanced age, and a deservedly high reputation as a teacher, practitioner, and citizen. He will carry with him, in his retirement, the good wishes of the whole profession.

---

LONDON LANCET.—This important monthly for September is promptly on our table, filled with its usual complement of interesting and profitable reading.

---

LONDON MEDICAL TIMES AND GAZETTE.—By way of Montreal, we have received the June and July numbers of this interesting and important foreign medical journal. Like the *Lancet*, it is filled with original lectures, communications, and practical matter gathered from the societies, hospitals, and medical institutions of the metropolis of Great Britain.

---

BRAITHWAITE'S RETROSPECT.—The semi-annual volume of this compend of foreign medical literature for July was promptly issued as usual, and will be found as interesting and profitable as any of its predecessors.

---

THE MECHANICAL TREATMENT OF ANGULAR CURVATURE OF THE SPINE, OR POTT'S DISEASE. BY CHARLES FAYETTE TAYLOR, M.D., of New York.

This is a neatly printed pamphlet of 48 pages, containing an essay read before the New York State Medical Society, at its fifty-third annual meeting in 1863. It is a plain, practical, and interesting essay, which will well repay perusal.

---

PERMANGANATE OF POTASH AS A DISINFECTANT.—Dr. Ploss, speaks in the highest terms of the disinfecting power of this substance. It effectually removes all smell from the most stinking suppurating sores and discharges. Most remarkable results of this kind have followed its injection, repeated several times a-day, in cases of cancer of the uterus—half a drachm to eight ounces of distilled water being a good proportion. In the case of open wounds and ulcers, all the dressings covering them should be moistened with the solution. No means succeeds more rapidly than this in removing the disagreeable smell of the hands after the performance of autopsies, for which purpose a stronger solution (ʒss. ad ℥j.) may be employed. It is far superior to chlorine in its effects, which are not, as is the case with that substance, fugitive. For this reason it is a superior prophylactic, applied to the hands of accoucheurs, to chlorine in puerperal fever. In ozœna, it is strongly to be recommended, the solution (℈ss. ad ℥vii.) being introduced into the nares by means of a caoutchouc syphon. In bad smells of the mouth, resulting from carious teeth, it is an admirable means, a little cotton wool being moistened in a weak solution. Finally, the permanganate is to be recommended as a wash for stinking feet. This remedy deeply stains linen it comes in contact with, but the spots may be removed by means of the sulphate of iron.—*American Druggists' Circular.*

---

Liquor Bismuthi.—Most practitioners, we believe, agree in opinion as to the special value of bismuth in painful affections of the stomach, however much they may differ as to the nature of the pathological conditions giving rise to these very common painful states of the organ. We have hitherto been confined to two preparations,—the trisnitrate and the carbonate. Both these are insoluable powders, bulky and inconvenient, inasmuch as a sufficient dose cannot be made into one or two pills.

Mr. Schacht, of Clifton, has succeeded in preparing a solution of bismuth, which is uniform in composition, stable, miscible with water or other fluids without precipitation, and efficient in small doses. It appears to us a most convenient form for the exhibition of the remedy. This solution is quite transparent, with a slight alkaline reaction; and although it contains only eight grains of oxide of bismuth in an ounce, a fluid drachm for a dose is found to be equivalent to a full dose,—fifteen or twenty grains,—of the insoluable trisnitrate. Mr. Schacht states, that before he ventured to introduce the Liquor Bismuthi to the profession generally, he had its efficacy tested by four years' experience of the practitioners of his neighborhood.

Dr. S. Martyn, Senior Physician to the Bristol General Hospital, says, "For several years past, in prescribing bismuth, I have used almost exclusively the solution made by Mr. Schacht, of Clifton. It has seemed to me to act better than the old forms. I find it allays pain in acute irritability of the stomach

(without nausea or much acidity,) and especially in that which remains after ulceration. In hospital practice, I have observed remarkable ease produced by it when given quite alone,—i. e., simply diluted with water; while it was always more satisfactory to me to use a fluid of agreeable taste than a cumbrous powder, imperfectly suspended, and not of very certain composition."

Mr. Schacht's Liquor Bismuthi is unquestionably an "improved preparation," and will assuredly be adopted by the profession.—*London Lancet.*

---

THE MEDICAL AND SURGICAL HISTORY OF THE WAR.—Dr. J. Janvier Woodward, of Philadelphia, Assistant-Surgeon U.S. A., is now writing a "Medical History of the War." One volume, which includes Dr. Woodward's experience on the Peninsula with the Army of the Potomac, and his practice in the Surgeon-General's office, up to July, 1862, has been completed. The next volume, which will bring the medical history of the war up to July, 1863, will be completed some time during the coming year. The lithographic plates for this work are now being executed in Philadelphia, and will cost $50,000.

Besides this work, Dr. Woodward has nearly completed a work on "The Camp Fevers of the Army," which will soon be issued. It appears that fevers prevailed to a much greater extent in the Eastern than in the Western armies. Dr. Woodward is the author of the "Hospital Manuel" adopted by the Surgeon-General and used in all the United States Army General Hospitals throughout the country.

The "Surgical History of the War" is being written by Dr. Brinton, Assistant-Surgeon, U.S.A., and will soon be completed up to July, 1862, to correspond with the work of Dr. Woodward. Dr. Brinton's work will be issued in annual volumes. The plates for this work also cost $50,000, and are in progress in Philadelphia.

---

ACTION OF SOLAR RAYS ON EXPOSED INTESTINES.—At the meeting of the Academy of Medicine of Paris, on the 16th ult., M. Sappey read a report on a case of severe wound of the abdomen. The patient was a shepherd boy, aged eleven, who was gored by a bull, and to such an extent that the stomach, spleen, and a large portion of the intestines were protruding. Being far from any help, the poor boy lay for two hours with the viscera just mentioned exposed to the action of a boiling sun. Dr. Patry found the patient in this pitiable state; and, by dint of care and perseverance, the boy recovered. His medical attendant seized, however, upon this opportunity to watch the mechanism of vomiting, and found that the phenomena succeeded each other in the following manner:—Contraction of the diaphragm,—vermicular contraction of the stomach, commencing at the pylorus and running from the latter to the cardiac orifice,—forcing of the liquids contained in the stomach towards the œsophageal opening,—energetic contraction of the œsophagus,—involution of the stomach at every effort,—dilatation of the cardia under the influence of the longitudinal fibres of the œsophagus,—finally, filling of the latter canal by the liquids coming from the stomach, and vomiting.—*Lond. Lancet.*

